# Free-Form Deformation Approach for Registration of Visible and Infrared Facial Images in Fever Screening †

**DOI:** 10.3390/s18010125

**Published:** 2018-01-04

**Authors:** Yedukondala Narendra Dwith Chenna, Pejhman Ghassemi, T. Joshua Pfefer, Jon Casamento, Quanzeng Wang

**Affiliations:** 1 Center for Devices and Radiological Health, U.S. Food and Drug Administration, Silver Spring, MD 20993, USA; cyndwith@gmail.com (Y.N.D.C.); Pejhman.Ghassemi@fda.hhs.gov (P.G.); joshua.pfefer@fda.hhs.gov (T.J.P.); Jon.Casamento@fda.hhs.gov (J.C.); 2 Department of Electrical and Computer Engineering, University of Maryland, College Park, MD 20740, USA

**Keywords:** thermal imaging, fever screening, temperature measurement, canthi detection, multi-modality image registration, free form deformation, Demons algorithm, cubic B-spline algorithm

## Abstract

Fever screening based on infrared (IR) thermographs (IRTs) is an approach that has been implemented during infectious disease pandemics, such as Ebola and Severe Acute Respiratory Syndrome. A recently published international standard indicates that regions medially adjacent to the inner canthi provide accurate estimates of core body temperature and are preferred sites for fever screening. Therefore, rapid, automated identification of the canthi regions within facial IR images may greatly facilitate rapid fever screening of asymptomatic travelers. However, it is more difficult to accurately identify the canthi regions from IR images than from visible images that are rich with exploitable features. In this study, we developed and evaluated techniques for multi-modality image registration (MMIR) of simultaneously captured visible and IR facial images for fever screening. We used free form deformation (FFD) models based on edge maps to improve registration accuracy after an affine transformation. Two widely used FFD models in medical image registration based on the Demons and cubic B-spline algorithms were qualitatively compared. The results showed that the Demons algorithm outperformed the cubic B-spline algorithm, likely due to overfitting of outliers by the latter method. The quantitative measure of registration accuracy, obtained through selected control point correspondence, was within 2.8 ± 1.2 mm, which enables accurate and automatic localization of canthi regions in the IR images for temperature measurement.

## 1. Introduction

Mitigation of the threat of infectious pandemics such as the Ebola virus disease may be possible through mass screening in the field or at public transportation centers such as airports. Fever screening based on non-contact infrared (IR) thermometers (NCITs) and IR thermographs (IRTs) represents the only currently viable approaches. While NCITs are more commonly used for fever screening, IRTs may be more effective if implemented according to the recommendations of a recently published standard [1]. For example, this standard indicates that IRT measurements should be performed with an accurate blackbody in the image as a reference and that the preferred tissue sites for imaging—regions medially adjacent to the inner canthi (termed as canthi regions)—provide the most accurate estimates of core body temperature. However, it is more difficult to accurately identify canthi regions from IR images than from visible images, as the latter provides a greater number of exploitable features. Therefore, in development of rapid, automated devices for identification of the canthi regions within facial IR images, the use of multi-modality image registration (MMIR) of simultaneously captured visible and IR images will likely produce optimal results.

MMIR of visible and IR images is well studied in the literature for facial recognition, image fusion and other applications [2,3,4,5,6,7,8,9]. However, fever screening requires high registration accuracy at the canthi regions, instead of the whole face. To the best of our knowledge, no MMIR study focused on the canthi regions has been performed. In the case of visible and IR facial images, intensity-based similarity metrics—which are widely used in medical imaging—cannot be directly applied because these two image types do not exhibit correlation in pixel intensities. However, certain common features like high curvature, line intersections, strong edges, and structure contours within the images are consistent in both visible and IR images and therefore can be used to obtain edge maps for improving the registration. The relationship between these images can be modeled using an affine transformation. Constrained by bones and muscles, human faces are non-planar and non-rigid, which limits the accuracy of affine transformations. Accuracy of affine transformation can be improved through free form deformation (FFD) transformation since FFD can generate smooth transform and avoid unnatural deformation. 

In this paper, we studied the methods for MMIR of visible and IR facial images to enable the accurate detection of the canthi regions. We used a two-step coarse-fine registration method for visible and IR images. The coarse registration step used the affine transformation, and the fine registration step was based on an FFD model using the edge maps. The edge maps near the eyes are significant common features preserved in both the visible and the IR facial images [5,6,10]. These features served as the reference for estimating the FFD transformation. Two FFD models widely used in medical image registration based on the Demons and cubic B-spline algorithms were qualitatively compared.

### Image Registration

Image registration involves spatial alignment of images through transformations and can be formulated as a spatial transformation that defines correspondence between two images. Depending on the application, a registration algorithm can be decomposed into three components: a similarity metric [11], a transformation model [12,13,14], and an optimization method [14,15,16].

**Figure 1:**
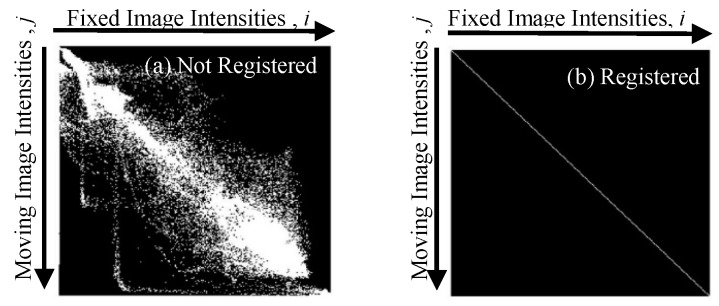
Joint histograms for measuring registration accuracy using the MI metric: (**a**) low accuracy, high entropy; (**b**) high accuracy, low entropy [19].

*Similarity Metrics* determine the accuracy, robustness, and flexibility of a registration algorithm. They can be broadly classified into landmark-based and intensity-based metrics [11]. A landmark-based metric can be used to find transformation using unique landmarks in the reference and moving images. An intensity-based metric works directly with pixel intensity levels and offers flexibility and robustness [15], which makes it suitable for most image registration applications. In the case of different modalities, the intensity-based similarity metric based on correlation (e.g., normalize cross correlation (NCC)) or statistic measures (e.g., entropy-based mutual information (MI)) of pixel values are well suited for MMIR. Intensity-based metrics widely used for medical image registration, include sum of squared difference (SSD), NCC, and MI [11]. The SSD similarity metric is the simplest similarity metric for image registration. It can be calculated as:

(1) $$I_{SSD}=\sum^{\text{​}}{\left[I_{reg}\left(x,y\right)-I_{ref}\left(x,y\right)\right]}^{2}$$

where $I_{SSD}$ is a measure of the SSD metric, $I_{ref}$, and $I_{reg}$ are the intensity matrices of the reference image and the transformed moving image (i.e., the registered image), respectively. $I_{SSD}$ is often used for images with similar modality, which gives an optimal solution if the images are aligned with white Gaussian noise. The NCC similarity metric is a more general similarity metric. It assumes a linear intensity relationship between images $I_{ref}$ and $I_{reg}$ and can be expressed as:

(2) $$I_{NCC}=\frac{\sum^{\text{​}}\left(I_{ref}\left(x,y\right)-\mu_{ref}\right)\left(I_{reg}\left(x,y\right)-\mu_{reg}\right)}{\sqrt{\sum^{\text{​}}{\left(I_{ref}\left(x,y\right)-\mu_{ref}\right)}^{2}\sum^{\text{​}}{\left(I_{reg}\left(x,y\right)-\mu_{reg}\right)}^{2}}}$$

where $\mu_{ref}$ and $\mu_{reg}$. are the mean image intensities of the reference and registered images, respectively. The MI similarity metric for image registration was first proposed by Woods et al. [17]. In the case of MMIR, regions with different intensity levels in an image would correspond to similar regions in another image that also contain similar number of intensity levels (maybe of different values). Ideally, the correspondence between these intensity levels might not change significantly across either of these images. Hill et al. [18] proposed a registration method by constructing a joint histogram, which is defined as a two-dimensional plot showing combinations of intensity levels (Figure 1). The value at location (*i*, *j*) in a joint histogram represents the number of pixels whose intensity level is *i* in one image and *j* in the other. A joint histogram shows decreased dispersion as registration accuracy increases. Shannon entropy (referred as entropy) is used as a metric to measure the dispersion in a joint histogram. If *X* = {*x*_1_, *x*_2_, …, *x_n_*} is a finite discrete set and each element has probability *p_i_*, the entropy of *X* is given by:

(3) $$H\left(X\right)=-\overset{n}{\underset{i=1}{\sum }}p_{i}\log_{2}p_{i}.$$

Entropy only depends on the distribution of the random variables. This definition of entropy can be extended to images, where the probability distribution function is constructed using the histogram distribution of the pixel values in the image. Entropy of a joint histogram decreases as the alignment of images increases as shown in Figure 1. The MI metric *I_MI_ (A*; *B)* can be defined as [11]:

(4) $$I_{MI}\left(A;B\right)=H\left(A\right)+H\left(B\right)-H\left(A,B\right)$$

where H(A), H(B), and H(A,B) are entropies of image A, image B, and joint histogram, respectively. The maximization of *I_MI_* can be achieved by minimizing the entropy of the joint histogram. Hence, the problem of registration is converted into an optimization problem involving maximization of the MI metric using different transformations. The MI metric is widely used for MMIR.

A *Transformation Model* defines the relationship between the coordinates of two images. Let *I_ref_*, *I_mov_*, and *I_reg_* denote the reference, moving, and registered images, respectively, the goal is to find a transformation matrix *T*(*x*, *y*) (or equivalently its inverse) which provides a mapping from *I_mov_*(*x*, *y*) to *I_reg_*(*x*, *y*) so that *I_ref_* and *I_reg_* have sufficient similarity. Transformation models can be classified as linear transformations (e.g., rigid, affine, projective) and non-linear transformations (e.g., curved (or elastic), FFD) [20,21]. Linear transformations are global (i.e., warping the whole image) [20] and may include translation, rotation, scaling, reflection, shear, and projection. Simple planar surfaces can be modeled through translation, rotation, scaling, and shear, which together define an affine transformation. Affine transformation preserves the parallelism of lines, but not their lengths or angles. It extends the degrees of freedom for the rigid transformation with scaling factor and shear. 

In many cases, MMIR uses a non-linear transformation by locally displacing a moving image. FFD transformation algorithms are widely used for MMIR. They can be broadly classified into parametric and non-parametric algorithms. The parametric algorithms (e.g., B-spline algorithms [15]) are defined on a coarse grid of control points and contrast with non-parametric algorithms (e.g., Demons algorithm [12]) where a displacement vector is associated with every pixel in the moving image. Both the parametric and non-parametric transformations have many parameters and are computationally intense, which can be handled through a registration strategy based on a hierarchical Gaussian pyramid. In the case of parametric transforms, the displacement field at every pixel (*x*, *y*), *T*(*x*, *y*), is defined as a function of displacement vectors (*u*, *v*) in the neighborhood of coarse grid control points.

(5) T(x, y)=(x+f(u), y+f(v))

where *u* and *v* are vertical and horizontal displacement fields, and *f* is the weight of basis function used to define the transformation. Given a pair of images *I_ref_* and *I_mov_*, we wish to simultaneously recover *u* and *v*. The parametric algorithms use a combination of vectors to define the displacement at a general location in the image with nearer vectors having a greater influence. The inherent smoothness of cubic B-spline functions also defines smooth displacement vectors without the need for external smoothness constraints. In the case of non-parametric algorithms, an additional smoothness constraint on the transformation based on a weighted Gaussian function can be included. This smoothness constraint eliminates un-natural deformations in the transformation. 

An *Optimization* method tries to minimize (or maximize) the specified similarity metric over the search space of possible parameters for the transformation model. An effective optimization method must be reliable and quick to find the best possible parameters of the transformation model. Selection of an optimization method depends on the application, transformation model, time constraints, and required accuracy of the registration. In non-linear registration applications, the optimizer is more complicated as a non-linear transformation model has more parameters than a linear one. Many registration problems can be solved using a gradient-descent-based optimization method, with existing numerical solvers [15,16]. 

## 2. Implementation 

**Figure 2:**
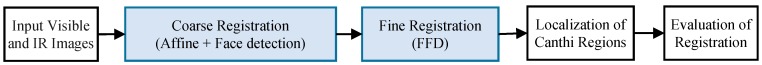
Block diagram of the two-step registration strategy.

**Figure 3:**
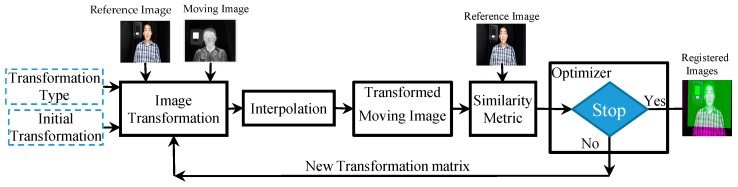
Block diagram of image registration.

To implement an automated temperature measurement, accurate IR-visible face registration for localization of the canthi regions is essential. We used a two-step registration strategy with coarse and fine registrations (Figure 2). The visible images were used as the reference images and the IR images were used as the moving images. The coarse registration was used for alignment and detection of facial images. The fine registration was used to improve the registration accuracy for accurate detection of the canthi regions. The coarse and fine registrations shared the same principle as shown in Figure 3. The registration algorithm was an iterative process, where we selected the transformation type and started with some initial estimate to transform the moving image. Interpolation was used during the transformation to estimate the pixel values at non-integer grid locations. The transformed image was compared with the reference image to generate the similarity metric. The regular step gradient descent optimizer in MATLAB (MathWorks, Natick, MA, USA) was used to adjust the variables for the transformation to minimize (or maximize) the similarity metric. 

The images of volunteers were captured using a visible camera (HD Pro Webcam C920, Logitech International S.A., Lausanne, Switzerland) and an IRT (8640 P-series, Infrared Cameras Inc., Beaumont, TX, USA). The IRT has 512 × 640 pixels. While the visible camera has 1920 × 1080 pixels, the compressed images only have 640 × 480 pixels. The field of view values were measured [22] to be 62° and 48° for the visible camera and 50° and 60° for the IRT, in vertical and horizontal directions respectively. The visible camera and IRT were placed at closest possible locations with the center-to-center distance of their optical lenses being 3 cm. An IR image was always taken simultaneously with a visible image to form an image pair. The image pairs from the visible camera and IRT were used to develop the registration method and quantitatively measure the registration accuracy. The accuracy of automatic temperature reading from the canthi regions with the two-step registration method described above was compared with the accuracy of temperature reading from the manually selected canthi regions.

### 2.1. Coarse Registration

The coarse registration was based on an affine transformation with a MI similarity matric, followed by face detection. The face detection algorithm was based on work by Viola and Jones [23], a cascade boosted classifier using Haar-like digital image features trained with positive and negative examples. The pre-trained frontal face classifier available with the MATLAB computer vision library was used to obtain the location and size of the face region [24]. The coarse registration algorithm was implemented in MATLAB using the Mattes MI algorithm [11]. In this algorithm, single intensity pixel/sample was drawn from images. The marginal and joint probability density function (PDF) was evaluated at discrete positions using samples of pixel intensities. The regular step gradient descent optimizer [25] was used for implementation of this algorithm. 

### 2.2. Fine Registration

We used fine registration to improve registration accuracy of IR-visible image pairs and to enable accurate localization of the canthi regions. Unlike the affine transformation [24,26] used for coarse registration, the fine registration uses FFD and needs to define a vector field for each pixel in the image. To model the local deformations of a face that are difficult to describe via affine transformations, we evaluated both the Demons and the cubic B-spline algorithms, which are widely used in medical imaging as an FFD model. 

*The Demons Algorithm* [12] proposes non-linear registration as a diffusion process, which introduces entities called Demons that exert forces according to local image characteristics. These forces are inspired from optical flow equations [13]. The basic idea of Demons algorithm for non-linear registration is that the reference image acts as a local force which moves pixels in the moving image to match the reference image. During each iteration, the moving image is transformed using the moving vector *dV =* (*dx*, *dy*) for each pixel as follows:

(6) $$dV^{\left(n+1\right)}=\frac{\left(I_{mov\;}^{\left(n\right)}-\;I_{ref}\right)\times \left(\nabla I_{ref}\right)}{\left(I_{mov}^{\left(n\right)}\;-\;I_{ref}\right)+\;\left|\nabla I_{ref}\right|}.$$

where $I_{ref}^{}$ represents the intensity of reference image, and $I_{mov}^{\left(n\right)}$ represents the intensity of the moving image at the n^th^ iteration. When *n* = 0, $I_{mov}^{\left(0\right)}$ represents the intensity of the original moving image. Gaussian filter is used to smooth the displacement fields, which enables noise suppression and preserves geometric continuity of the deformed image. The gradient of the reference image $\nabla I_{ref}^{}$ is computed only once during the iterations. Moreover, Demons algorithm assumes that the displacement vector is reasonably small or local. In some cases, such an assumption might be violated, which can be mitigated through a multi-scale approach that reduces the magnitude of these displacement vectors. For instance, the reference and moving images can be down-sampled to low-resolution images and the displacement fields at each stage can be up-sampled to a finer scale. Such a multi-scale approach also enables a large computational advantage for large image sizes. The Demons algorithm is widely used for similar modality images using the SSD error as the similarity metric [15,16]. We tried to reduce the differences between modalities by using common features in visible and IR images. We generated edge maps for visible and IR images using the canny edge detector for non-linear transformation. These edge maps emphasize the contour edges of face and show good similarity between visible and IR images [5,7]. The eye regions were used to predict the FFD for fine registration. As an iterative optimization method, effective stopping criteria needed to be defined. We used a tolerance criterion that stops the iterations if the mean square error (MSE) increases with the iterations or the threshold decreases for each iteration within a convergence tolerance. 

*Spline*-*based algorithms* are among the most common and important transformation models used for non-linear registration in medical imaging. Spline-based registration algorithms use control points in the moving image and a spline function that defines the transformation away from these points. They can be broadly divided into two types based on thin-plate splines and B-splines. Thin-plate splines have global influence on the transformation. In contrast, B-splines are only defined near the control points. Any perturbation in the control points influences only the neighborhood of that point and the deformation is defined by manipulating the underlying mesh of the control points. This makes B-spline-based registration a computationally efficient alternative to other non-linear registrations. Implementation of B-spline uses a uniform mesh of the control points such that each set of m × m pixels correspond to a single spline patch defined by four control points. The resolution of the control points defines the degrees of freedom and consequently the computational complexity. A large spacing of the control points allows modeling of global non-linear deformations, while a small spacing of control points allows modeling of highly local deformations. The displacement fields are estimated using two dimensional splines controlled by a small number of control points in the moving image. The B-spline basis function imposes an implicit smoothness on the motion field, without the need for additional smoothness constraints. The $u_{i}$ and $v_{i}$ defines the displacement vectors at each pixel in the *x* and *y* directions, respectively.

(7) $$u_{i}=u\left(x_{i},y_{i}\right)=\;\sum^{\text{​}}U_{i}w_{ij}$$

(8) $$v_{i}=v\left(x_{i},y_{i}\right)=\;\sum^{\text{​}}V_{j}w_{ij}$$

where $w_{ij}$ are called basis functions and are non-zero over a small interval. It emphasizes that the (*u*, *v*) are a known linear combination of ($U_{j}$,$\;V_{j}$) control points. We used this hierarchical registration with different control point mesh resolutions. The spacing between control points decreased at each level, which increases the resolution of the control point mesh. The horizontal and vertical control points halved in every step. The control point mesh at one level was refined by inserting new control points to create the control point mesh at the next level. Each control point mesh and associated spline-based FFD defined the transformation at each level of resolution. To avoid the overhead of calculating several B-spline FFDs separately, we represented the transformation by a single B-spline FFD whose control point mesh was progressively refined. Based on the order of basis function, the B-spline can be further classified into linear, quadratic, cubic, or higher-order B-spline registrations. The higher-order basis functions improve the registration accuracy at the cost of longer computation time. Cubic B-spline is quite widely used in medical image registration as it offers balanced tradeoff between registration accuracy and computation time [15,24]. Therefore, cubic B-Spline was used in this paper.

Similarity metrics of SSD and NCC were evaluated for the Demons and cubic B-spline algorithms, and the preliminary data (not listed in the paper to limit the paper length) showed that the SSD metrics was better than the NCC metrics. Therefore, all the fine registration data shown in this paper were based on the SSD metrics.

## 3. Results

We recruited 52 subjects for the study. We evaluated the registration accuracy in two approaches based on circular aluminum foil markers on 6 subjects and manually selected landmarks on 10 subjects. We also compared the temperature reading results on 36 subjects based on manual canthi selection and automatic canthi detection, respectively. Different subjects were used for different evaluation approaches and temperature reading study to avoid reduplicative information.

### 3.1. Registration Accuracy

**Figure 4:**
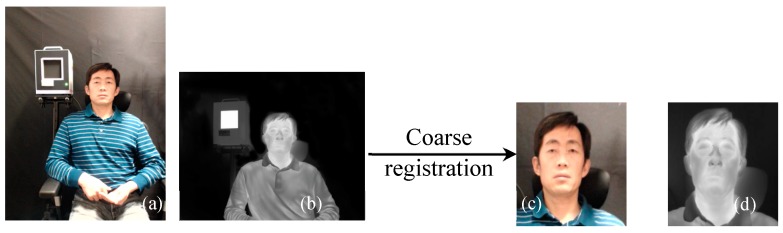
Visible (**a**,**c**) and IR (**b**,**d**) images before (**a**,**b**) and after (**c**,**d**) coarse registration.

**Figure 5:**
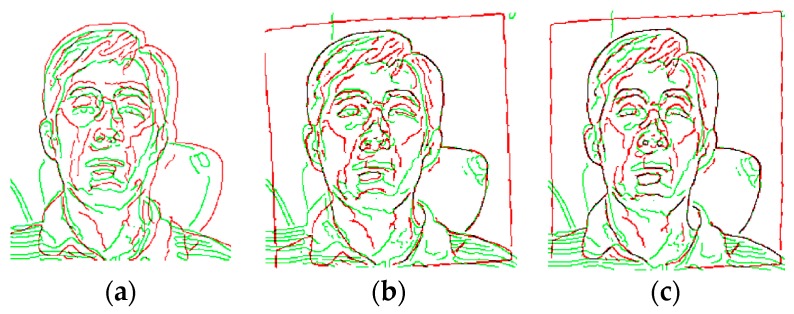
Edge map pairs view of registered visible (green) and IR (red) images with the (**a**) coarse, (**b**) coarse-Demons, and (**c**) coarse-spline methods.

The two-step coarse-fine registration strategy of visible and IR images was qualitatively evaluated. Figure 4 shows a pair of visible and IR images before and after the coarse registration. After coarse registration, the original images were cropped and only the face region in each image was used for the fine registration step. Figure 5 shows the registration results of the edge map pairs using coarse and coarse-fine registration methods. The edge maps were extracted from the Canny edge detector. The fine registration can be based on the Demons or the cubic B-spline algorithm. For simplicity, we call the two-step coarse-fine registration methods the coarse-Demons and coarse-spline methods, respectively. From Figure 5, prominent edge features like eyes, nose, and mouth with the coarse-Demons and coarse-spline methods have been better aligned compared to the coarse registration alone. 

**Figure 6:**
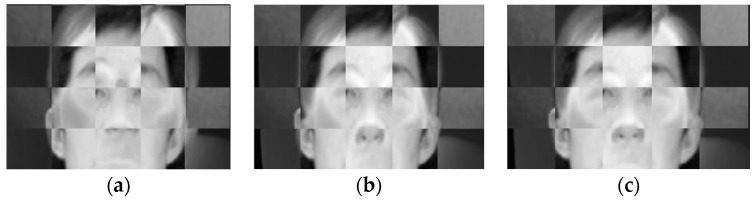
Checkered view of registered images using the (**a**) coarse, (**b**) coarse-Demons, and (**c**) coarse-spline methods.

Figure 6 shows corresponding image registration results viewed through superimposed checkerboard pattern of visible and IR images in gray scale. It can be observed that the eyes and nose are not accurately aligned with the coarse registration, whereas the coarse-Demons and coarse-spline methods show better alignment. This demonstrates that applying a non-linear registration algorithm improves accurate matching of face images compared to the coarse registration alone.

**Figure 7:**
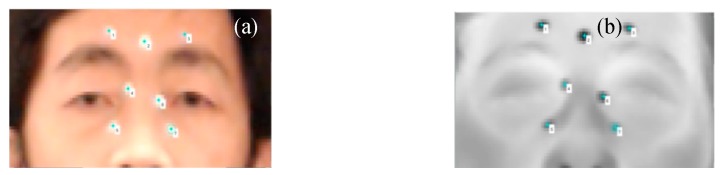
Aluminum Markers as control points for registration accuracy evaluation: (**a**) visible image; (**b**) IR image [19].

We quantitatively evaluated the two-step coarse-fine registration strategy of visible and IR images with markers attached to the subjects. Circular aluminum foil markers with a diameter of 7 mm were attached to different locations around the canthi regions of 6 volunteers to evaluate the registration accuracy. The markers and their correspondence in each set of visible and IR images (Figure 7) were manually selected to define control points. They were removed using the Spot Healing Brush Tool in Adobe Photoshop^®^ before the image registration process to avoid any registration bias caused by them. After the image registration, the spatial transformation obtained was applied to the control points for a direct registration without further optimization. The distances between the transformed marker locations on the moving image and their corresponding locations on the reference image were used for registration accuracy analysis. The registration accuracy was analyzed with both MSE and recall [7,26] values. After image registration, the MSE of distances between each pair of transformed markers in the visible and IR images was calculated as a qualitative performance metric. We used Medical Image Registration Toolbox (MIRT) [24] for the cubic B-spline registration, to compare the coarse-spline method with the coarse and coarse-Demons methods. The registration MSE based on markers (Table 1) for the coarse registration was 5.0 ± 1.6 pixels on 6 subjects. It improved to 3.6 ± 1.5 pixels with the coarse-Demons method. The coarse-spline method only slightly improved the accuracy than the coarse method. The scale factor of images was measured to be ~0.8 mm/pixel. Therefore, error in localizing the canthi regions with the coarse-Demons method was converted to 2.8 ± 1.2 mm. We evaluated the uncertainty in manual selection of aluminum markers by repeating the manual selection process for 30 times on the same set of images. This gave us a statistical measure of standard deviation in manual selection to be 0.5 mm.

**Figure 8:**
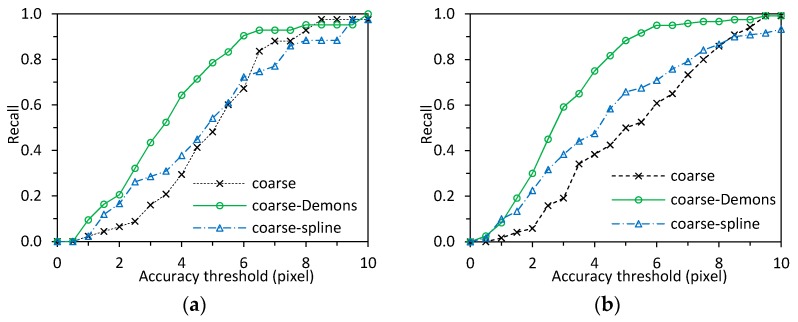
Recall graphs showing image registration accuracy of the coarse, coarse-Demons and coarse-spline models: (**a**) Markers in the face region as the control points based on the subjects in Table 1; (**b**) Landmarks around the eye region as the control points based on the subjects in Table 2.

The registration accuracy was also analyzed with the recall parameter that is defined as a fraction of the true positive correspondences to the ground truth. The recall values on all marker pairs were computed (Figure 8). The true positive correspondence was counted when the pair falls within a given accuracy threshold of the Euclidean distance between points in the registered image and the corresponding points in the reference image. The recall graphs plot the recall values against different threshold values, where the recall values are defined as the ratio of the number of markers within a threshold to the total number of markers. Figure 8a reports the recall values against the threshold values using registration methods of coarse, coarse-Demons, and coarse-spline, and with markers in the face region as the control points. The recall curves were based on average values from the subjects in Table 1. From Figure 8a, the algorithms using the coarse-Demons method outperforms the other methods. 

**Figure 9:**
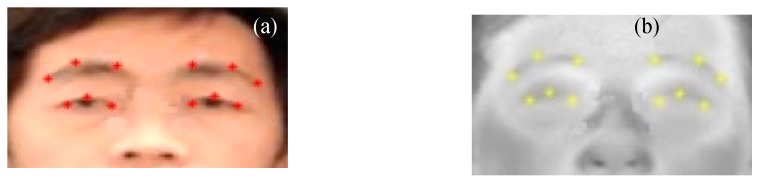
Landmarks as control points for registration accuracy evaluation: (**a**) visible image; (**b**) IR image.

We also evaluated the registration accuracy for canthi localization using manually selected landmarks in the eye region as the control points (Figure 9). The point correspondence on contours around eye region like eyes corners, eye brows, and pupil as ground truth, were identifiable in both the visible and IR images. The registration accuracy was analyzed with both MSE (Table 2) and recall (Figure 8b) values. The MSE of distance between each pair of landmarks in visible and IR images was 5.1 ± 1.9 pixels for the coarse registration on 10 individuals. It was improved to 3.2 ± 1.6 pixels after the Demons registration with the coarse-Demons method. The coarse-spline method showed slightly better accuracy than the coarse method. Figure 8b reports the recall values against the threshold values using registration methods of coarse, coarse-Demons, and coarse-spline. The recall curves were based on average values from the subjects in Table 2. As shown in Figure 8b, the recall plots for the course-Demons method (green plot) were consistently better than the recall plots for other methods for different threshold values. The coarse and coarse-spline methods had similar accuracy. Comparison of Figure 8a with Figure 8b showed that the marker and landmark methods agree with each other.

### 3.2. Canthi Temperature Measurement

**Figure 10:**
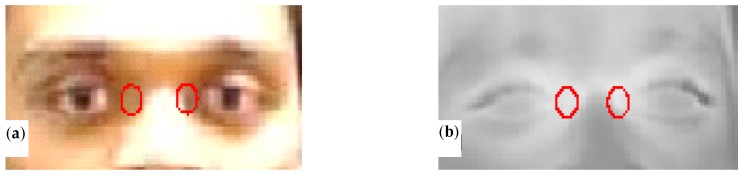
Canthi regions in (**a**) a visible and (**b**) an IR images [19].

The canthi regions are not susceptible to other factors (exertion, environment) because the blood in these regions is sufficiently supplied by the internal carotid artery and the heat loss is less than other regions such as the forehead. A recent study [25] and the IEC 80601-2-59 standard [1] indicate that the canthi regions (Figure 10) have the most stable temperature with good correlation to the core body temperature and thus are the best regions for fever screening. The canthi regions can be manually selected from a thermal image to read the temperature (manual approach). However, the manual approach is time consuming and lacks consistency. With the two-step coarse-Demons registration strategy we developed, the temperature at these regions can be quickly and automatically read (automatic approach). While Section 3.1 validates the accuracy of our two-step MMIR approach, this section addresses the MMIR approach based on clinical study data. We compared the automatic approach with the manual approach for reading inner canthi temperature. We used a Discriminative Response Map Fitting (DRMF)-based model [26] for facial key point detection as a tool to detect the canthi regions in the visible image. The detected canthi regions were mirrored to the IR image based on our coarse-Demons MMIR model, and the maximum temperatures (*Ta*) within the canthi regions were then automatically read from the IR image. At the same time, the canthi regions were manually selected from the IR image to read the maximum temperatures (*Tm*). The average values of the maximum temperatures from the left canthi (LC) and right canthi (RC) regions were used to compare the manual and automatic approaches.

**Figure 11:**
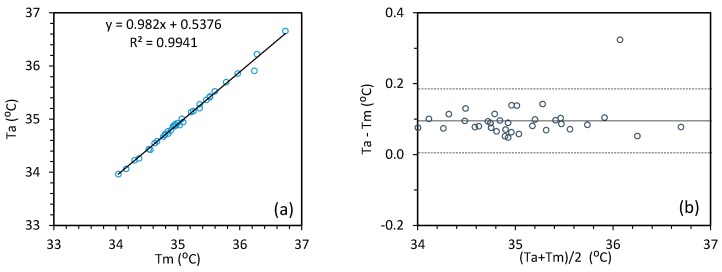
Automatic versus manual canthi temperature measurement (**a**) and the Bland-Altman plot (**b**).

We collected the canthi temperature data from 36 subjects through the manual and automatic approaches. We used Bland Altman plots (Figure 11) for combined graphical and statistical interpretation of the two measurement techniques. The plots were used to show the absolute differences between the two measurements against their mean (i.e., (*Ta* − *Tm*) versus (*Ta* + *Tm*)/2). The mean of absolute differences (Solid line in Figure 11) and the 95% limits (Dashed lines in Figure 11) of normal distribution (±1.96SD) are calculated for the estimation of inner canthi temperature from the manual and automatic approaches. We observed a difference between the automatic and manual methods of 0.10 ± 0.09 °C (mean ± 1.96SD).

## 4. Discussion

### 4.1. Image Registration Speed 

For real-time canthi detection, the image registration speed is important. The speed for the coarse and fine registrations on a consumer laptop (Dell laptop, Intel Core i7-6600 U CPU @ 2.6 GHz, 16 GB RAM) were 5.9 s and 2.9 s, respectively. The registration speed can be affected by many factors such as the computer speed, algorithm parameters, programing language, and image contents. While the registration speed is not the focus of this paper, we believe the registration speed can be improved by using a powerful computer, further optimizing the algorithm parameters, translating the Matlab codes to a faster language such as C/C++ codes, and adjusting the image contents.

### 4.2. Efffect of Image Quality on Registraton Accuracy 

**Figure 12:**
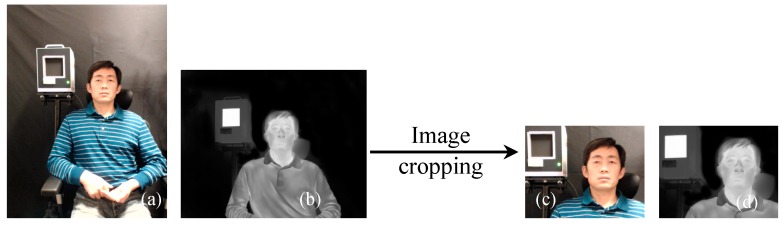
Sizes of images used for registration: (**a**) full size visible image, 640 × 480 pixels; (**b**) full size IR image, 512 × 640 pixels; (**c**) cropped visible image, 240 × 320 pixels; (**d**) cropped IR image, 240 × 320 pixels.

The optical performance of a camera, and thus the image quality, might affect the registration accuracy. As mentioned in Section 2, the IR and visible images have 512 × 640 and 640 × 480 pixels, respectively, as shown in Figure 12a,b. For canthi-based temperature measurement, we are only interested in face and blackbody region of the input images. During the process of coarse-fine registration, the input images were first cropped to the face region during the coarse registration step (Figure 4). Theoretically, we should be able to get the same registration results if the region of interest around the face has the same number of pixels even though the total pixel numbers of the whole images are different. To evaluate this assumption, we cropped both the visible and IR images to 240 × 320 pixels (Figure 12c,d)—the minimum required sizes based on the IEC standard [1]—to simulate camera with smaller number of pixels, before performing image registration. Our results showed that registration of Figure 12c,d had the same accuracy as the registration of Figure 12a,b.

Theoretically, higher image resolution for the same scene will improve registration accuracy. However, a larger pixel number doesn’t necessary mean a higher image resolution. The image resolution is affected by the camera’s modulation transfer function [27], which is not the focus of this paper. The qualities of two images from two cameras with the same physical parameters (e.g., pixel number, field of view, focal length) might be different, which might in turn result in different registration accuracies.

### 4.3. Effects of Other Factors on Registration Accuracy

While the data in this paper showed that the coarse-Demons registration was statistically more accurate for the current imaging system, the actual registration approach and parameters should be optimized and evaluated for a different imaging system to achieve optimum registration accuracy. Observed results showed that the edge detection algorithm had a significant impact on registration accuracy. The cubic B-spline registration showed degradation in performance due to edge map outliers. If the edge detection is improved through customized pre-processing, the accuracy of cubic B-spline registration might be improved for a specific IRT system. 

In our study, the visible camera and IRT were placed at the closest possible locations with the center-to-center distance of their optical lenses being 3 cm. If these two cameras can be closer so that they will see the subject from the same angle, the image registration should be more accurate. The intensity and uniformity of the illumination can also affect the registration. Non-uniform and too low/high illumination might deteriorate the registration accuracy. 

## 5. Conclusions

In this paper, we proposed a system that uses a two-step coarse-fine registration method for visible and IR face images for body temperature measurement using canthi regions. We evaluated two widely used FFD algorithms—the Demons algorithm and the cubic B-spline algorithm—as the second step of a coarse-fine registration method. The algorithms used edge maps to improve registration accuracy. The registration accuracy of the coarse-Demons and coarse-spline methods were qualitatively compared using MSE values and recall graphs from markers and landmarks. The results show that the coarse-Demons method outperformed the coarse-spline algorithm, likely due to overfitting of the outliers by the latter method. The quantitative measure of registration accuracy was within 2.8 ± 1.2 mm, which enables accurate and automatic localization of the canthi regions in IR images for temperature measurement. We used the coarse-Demons method for registration followed by canthi detection to read the temperature at the canthi regions from IR images. The temperature readings from the proposed automatic method were compared with the readings from the manual method through Bland Altman analysis. The comparison showed a difference of 0.10 ± 0.09 °C (mean ± 1.96SD) for the automatic and manual temperature readings.

## Figures and Tables

**Table 1:** Registration Matching Error (Markers in the face region as control points), pixels.

Methods	Coarse	Coarse—Demons	Coarse—Spline
Sub. M1	5.8	4.0	2.4
Sub. M2	3.4	4.4	4.6
Sub. M3	3.4	1.5	5.0
Sub. M4	4.0	2.2	6.1
Sub. M5	6.1	3.5	5.7
Sub. M6	7.3	5.8	5.9
Mean	5.0	3.6	4.9
SD	1.6	1.5	1.3

SD: Standard Deviation. Sub.: subject.

**Table 2:** Registration Matching Error (Landmarks around the eye region as control points), pixels.

Methods	Coarse	Coarse—Demons	Coarse—Spline
Sub. L1	7.6	5.2	2.1
Sub. L2	2.4	2.1	2.6
Sub. L3	3.4	4.4	4.2
Sub. L4	4.7	3.5	7.3
Sub. L5	5.4	6.4	4.4
Sub. L6	3.5	1.8	3.1
Sub. L7	8.5	2.3	6.0
Sub. L8	3.7	2.2	4.9
Sub. L9	6.6	1.6	6.3
Sub. L10	5.6	2.1	4.8
Mean	5.1	3.2	4.6
SD	1.9	1.6	1.7
